# Complete genome sequence of *Arthrobacter globiformis* bacteriophage Powelldog

**DOI:** 10.17912/micropub.biology.001920

**Published:** 2025-12-19

**Authors:** Zeel Desai, Nathaniel Foret, Skylar L. Godwin, Harrison B. Huffmon, Joseph Y. Kim, Lily Y. McGuirt, Shamanasia A. Richardson, Jessica Robles Gonzalez, Daune Signorelli, Heven Siyum, Anngiselle Vallejo, Sarah R. Wigren, Sharon K. Bullock, Michelle B. Pass, Ellen M. Wisner, Tonya C. Bates

**Affiliations:** 1 Department of Biological Sciences, University of North Carolina at Charlotte, Charlotte, North Carolina, United States

## Abstract

Bacteriophage Powelldog, assigned to cluster AW, was isolated from dog park soil in Charlotte, NC, using the bacterial host *Arthobacter globiformis* NRRL B-2880. The genome is 55541 bps, containing 91 putative protein-coding genes. All genes are transcribed unidirectionally and the genome, interestingly, encodes a putative major capsid protease fusion protein.

**Figure 1. The Powelldog genome map:**
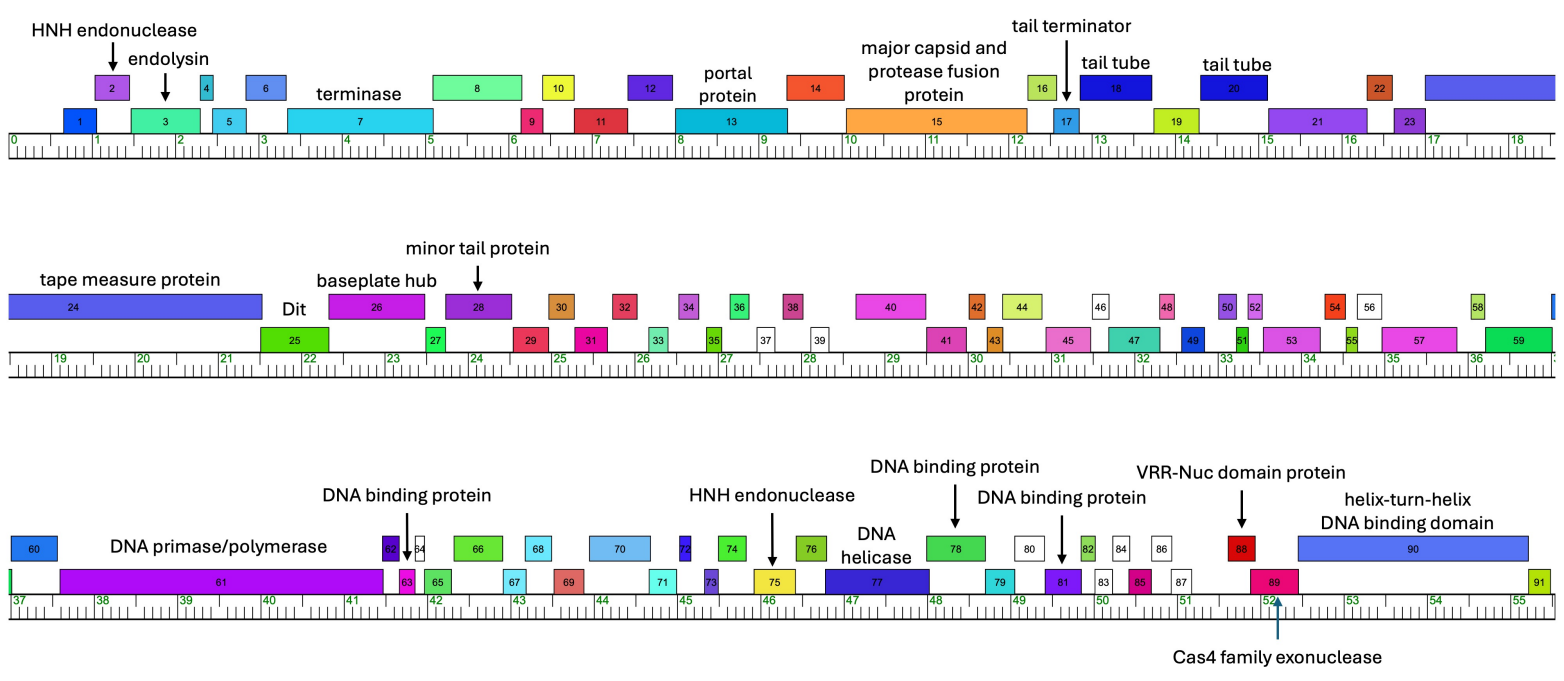
Putative genes are indicated with a box, with the number inside indicating the corresponding gene number in the genome. The base pair location is indicated by the ruler, with each hatch mark representing 100 base pairs. Powelldog has all putative genes transcribed unidirectionally, as shown with all boxes above the ruler. The orphams are indicated with white boxes. Maps were generated using Phamerator with database Actino_Draft_596 (Cresawn et al. 2011).

## Description

Bacteriophages are highly abundant viruses that infect bacteria and are actively being developed as therapeutics for treating antibiotic-resistant bacterial infections (Kasman & Porter 2022). Importantly, *Arthrobacter* strains have been implicated in clinical cases (Busse et al. 2015). We present the annotated genome sequence of the bacteriophage Powelldog.

Powelldog was isolated from soil collected near a dog park in Charlotte, North Carolina, USA (GPS coordinates: 30.29016 N, 80.74992 W). The soil sample was washed in PYCa (peptone, yeast extract, calcium) liquid medium, the wash filtered (0.22 um pore size), and the filtrate inoculated with *Arthrobacter globiformis* NRRL B-2880 and allowed to incubate at 30˚C for 48 hours. The resulting culture was then spun and refiltered before the filtrate was plated in PYCa top agar with *A. globiformis* and incubated at 30˚C to observe for the presence of phage via plaque formation (Zorawik et al. 2024). Powelldog formed tiny, clear plaques 0.50 - 1.1 mm (n=10) in diameter, and was purified through two rounds of plating.

Genomic DNA was isolated from a lysate of Powelldog using the Promega Wizard DNA Cleanup kit, prepared for sequencing using the NEB Ultra II Library kit, and sequenced using an Illumina NextSeq 1000 XLEAP-P1 kit. This yielded 2,829,918 100-base single-end reads, generating 4,839-fold coverage. Reads were trimmed with Cutadapt 4.7 (using the option: –nextseq-trim 30) and filtered with Skewer 0.2.2 (using the options: -q 20 -Q 30 -n -l 50) prior to assembly, then assembled and checked for completeness using Newbler (v2.9) (Miller et al. 2010) and Consed (v29) (Gordon et al. 1998; Russell 2018), respectively, with default parameters. This resulted in a genome that is 55,541 base pairs long and ends in a 9 bp long 3’ single-stranded overhang (CGCCGCTCC). The genome has a 51.7% GC content, which falls within the GC content range of 47.6-70.4%, found in phages that infect A. *globiformis* NRRL B-2880 (Russell & Hatfull 2017). Powelldog was assigned to cluster AW due to sharing greater than 35% gene content similarity to other cluster members in the Actinobacteriophage database (Pope et al. 2017; Russell & Hatfull 2017).

Glimmer v3.02 (Delcher et al. 2007) and GeneMark v2.5 (Besemer and Borodovsky 2005) were used to auto-annotate the genome. DNA Master v5.23.6 (cobamide2.bio.pitt.edu), Phamerator using Actino_draft database v596 (Cresawn et al. 2011), Starterator v596 (http://phages.wustl.edu/starterator/), BLASTp v2.16.0 using the Actinobacteriophage and NCBI non-redundant database (Altschul et al. 1990), DeepTMHMM v1.0.42 (Hallgren et al. 2022), and HHPred (Söding 2005) using PDB_mmCIF70_30_Mar, Pfam-A_v37, NCBI_Conserved_Domains (CD)_v3.19, and UniProt-SwissProt-viral70_3_Nov_2021 were used to verify the genes, their starts, and their functions. Default settings were used for all software. Ninety-one putative genes were found, of which sixty genes could not be assigned a function, and no tRNAs were identified using tRNAscanSE 2.0 (Lowe & Eddy 1997).

Powelldog, like the other 20 members of the *Arthrobacter* phage cluster AW to-date, has all identified genes transcribed unidirectionally. Assigned putative functions include multiple structure and assembly proteins. A frameshift typical of phage genes encoding the tail assembly chaperones could not be identified in Powelldog. Other notable functions include an endolysin and DNA metabolism functions such DNA helicase and Cas4 family exonuclease. Powelldog contained 10 orphams, which are genes that do not have known homologs (Figure 1; Cresawn et al. 2011). No immunity repressor or integrase functions could be identified, suggesting Powelldog is unlikely to establish lysogeny.

**Nucleotide sequence accession numbers**

Powelldog is available at GenBank with Accession No. PV876981 and Sequence Read Archive (SRA) No. SRX27501636.
